# Sclerostin and OPG/RANK-L system take part in bone remodeling in patients with acromegaly

**DOI:** 10.3389/fendo.2024.1472680

**Published:** 2024-12-17

**Authors:** Jowita Halupczok-Żyła, Aleksandra Jawiarczyk-Przybyłowska, Marek Bolanowski

**Affiliations:** Department and Clinic of Endocrinology, Diabetes and Isotope Therapy, Wroclaw Medical University, Wrocław, Poland

**Keywords:** acromegaly, sclerostin, osteoprotegerin, RANK-L, IGF-1, GH, BMD

## Abstract

**Introduction:**

Acromegaly is a disease characterized by enhanced bone turnover with persistently high vertebral fracture risk. Sclerostin is a glycoprotein, which acts as an inhibitor of bone formation and activates osteoclast-mediated bone resorption. The osteoprotegerin (OPG)/receptor activator for the nuclear factor κ B ligand (RANK-L) system is crucial for controlling bone metabolism.

**Objective:**

The study aimed primarily at evaluating sclerostin, OPG, and RANK-L concentrations in patients at different stages of acromegaly activity. The secondary aim was to identify an association of sclerostin with the OPG/RANK-L system and bone mineral density (BMD).

**Materials and methods:**

The study enrolled 126 patients aged 40 to 80 years, including 72 patients with acromegaly and 54 controls (CG). The acromegaly patients were further classified into the following subgroups: active acromegaly (AA), controlled acromegaly (CTA), and cured acromegaly (CA). Blood samples were taken from the participants to measure sclerostin, OPG, RANK-L, growth hormone (GH), and insulin-like growth factor-1 (IGF-1). Dual-energy X-ray absorptiometry was performed at the lumbar spine and hip.

**Results:**

Significantly lower sclerostin concentrations were observed in acromegaly patients compared with CG (AA, CTA, CA, CTA+CA, AA+CTA+CA vs CG; p < 0.001). Significant differences in OPG concentrations were revealed between the following groups: CTA vs CA (p=0.002), CTA vs CG (p<0.001), CTA+CA vs. CG (p<0.001), and AA+CTA+CA vs. CG (p<0.001). There were no significant differences in RANK-L concentrations between studied groups, regardless of the adopted classification (p>0.05). There were no statistically significant correlations between sclerostin and GH/IGF-1 or BMD. In the AA+CTA+CA group, there was a statistically significant positive correlation between SCL and OPG concentrations (r=0.271; p=0.022). A significant negative correlation between SCL and RANK-L was found in the AA group (r=-0.738; p=0.046).

**Conclusions:**

Patients with acromegaly have lower sclerostin concentrations than healthy controls, which may be a result of a compensatory mechanism to increased bone loss. The influence of the GH/IGF-I axis on bone remodeling may be mediated in part by the OPG/RANK-L system. The interaction between SCL and OPG/RANK-L system in acromegaly should be further elucidated.

## 1 Introduction

Acromegaly is a rare disease caused by excessive secretion of growth hormone (GH) and its peripheral mediator, insulin-like growth factor-1 (IGF-1). As a result of chronically increased GH and IGF-1 secretion, numerous systemic complications develop, including those related to the osteoarticular system (1, 2).

GH and IGF-1 play an important role in the regulation of bone metabolism. Before puberty, GH promotes bone growth in length, while in young adults it is responsible for peak bone mass formation. After that, it regulates bone turnover, helping to maintain bone mass. The anabolic effects of GH and IGF-1 result from the stimulation of proliferation and differentiation of osteoblasts and the formation of the extracellular matrix. On the other hand, GH and IGF-1 stimulate the resorptive activity of osteoclasts (3). Excessive secretion of GH and IGF-1 in acromegaly causes increased bone turnover, which is reflected in higher concentrations of bone formation and bone resorption markers (4, 5). After successful treatment, these parameters are normalized (6). Histomorphometric studies in patients with the active form of the disease confirm the occurrence of disturbances in the bone structure related to accelerated bone turnover (7). Commonly, acromegaly is considered to be one of the causes of secondary osteoporosis, however, patients also have normal bone mineral density (BMD) values or even higher values compared to the control group (3, 4). Studies conducted in recent years indicate that both women and men with acromegaly have an increased incidence of vertebral fractures, even despite normal BMD (5, 7).

Sclerostin (SCL), a glycoprotein mainly synthesized by osteocytes, plays an important role in bone metabolism (8, 9). This protein in humans is encoded by the SOST gene, located on the long arm of chromosome 17 (locus q12-q21) (10). SCL inhibits bone formation by acting antagonistically to the classical (canonical) Wnt signaling pathway. In addition, it stimulates osteoclastogenesis and osteoclast activity and is also involved in the process of osteocytic osteolysis (8, 11–15). Antibodies against SCL are expected to be of use in the anabolic treatment of osteoporosis and other diseases associated with low bone mass and increased risk of fractures (9, 12, 13). Initially, it was believed that anti-SCL antibodies could be a potential therapeutic option in treating bone complications, as well as in patients with acromegaly (16, 17). However, further research did not confirm this assumption. Data on the role of SCL in regulating bone metabolism in patients with acromegaly is limited and provides conflicting results (16–21).

The osteoprotegerin (OPG), receptor activator for nuclear factor κB ligand (RANK-L) and receptor activator for nuclear factor κB (RANK) play a key role in bone tissue remodeling by regulating osteoclastogenesis (22). OPG, a glycoprotein that is a member of the tumor necrosis factor receptor (TNFR) superfamily is a soluble receptor for RANK-L. Its main function is to reduce the availability of RANK-L to the RANK receptor on osteoclasts, which leads to the inhibition of osteoclastogenesis (23–25). RANK-L is a member of the tumor necrosis factor (TNF) family, which after binding to the RANK receptor on the surface of osteoclast precursor cells and mature osteoclasts, triggers the signaling pathway, ultimately leading to the transcription of osteoclastogenesis-related genes (26). An *in-vitro* study showed that IGF-1 inhibits OPG expression and promotes RANK-L expression. Additionally, a 1-year administration of recombinant IGF-1 in women resulted in 20% decrease in OPG serum level. The authors suggest that IGF-1-related bone resorption takes place through the influence on OPG/RANK-L system (27). On the other hand, GH stimulates the expression and secretion of OPG from osteoblasts (28). To the best of our knowledge, there is no data in the literature focused on the association between sclerostin and OPG/RANK-L system in acromegaly depending on the disease activity.

The aim of the presented study was to determine SCL, OPG, and RANK-L concentrations in patients with acromegaly with regard to the disease activity and to evaluate the association between sclerostin concentrations and OPG/RANK-L system, GH, IGF-1, and BMD.

## 2 Materials and methods

The study was conducted on 126 subjects aged 40 to 80 years, including 72 patients with acromegaly (45 females and 27 males) and 54 controls (33 females and 21 males). All subjects were recruited from the Department of Endocrinology, Diabetes and Isotope Therapy, Wroclaw Medical University.

Based on clinical findings and hormonal evaluation (IGF-1, GH), the group of acromegaly patients was divided into three subgroups: AA – active acromegaly (5 females and 4 males), CTA – controlled acromegaly (23 females and 16 males) and CA – cured acromegaly (17 females and 7 males). The criteria used for active acromegaly were increased IGF-1 concentration (IGF-1 values above 1.3 × upper limit of normal (ULN) matched for sex and age) and/or no GH suppression below 1.0 ng/ml during 75 g oral glucose tolerance test (OGTT). Patients with normal IGF-1 concentration (IGF-1 values up to 1.3 × ULN matched for sex and age) were assigned to the CTA or CA groups (29). Patients with prior unsuccessful surgery and receiving long-acting somatostatin analogs were recruited to the CTA group. Patients who had undergone successful surgical treatment were allocated to the CA group. We recruited a control group (CG) of 54 sex- and age-matched patients with no clinical symptoms of acromegaly and normal IGF-1 (age- and sex-matched) and GH values. We analyzed the results of the study using three classifications. The first classification compared the patients with acromegaly (AA + CTA + CA) and the CG. The second was used to assess the differences between the AA group, both controlled and cured acromegaly (CTA + CA), and the CG. The last one was based on the degree of disease activity (AA; CTA; CA; CG).

In the AA group, 2 subjects received 30-40 mg of long-acting octreotide intramuscularly, and 2 patients received a dose of 120 mg of long-acting lanreotide, administered subcutaneously every 28 days. In the CTA group, 15 patients were on long-acting octreotide (10-40 mg/dose), 14 patients received 120 mg of long-acting lanreotide, and 10 subjects were treated with 20-60 mg/dose of long-acting pasireotide. Diabetes mellitus was diagnosed in 2 AA patients, 20 CTA patients, 3 CA patients, and 7 controls. Hydrocortisone replacement therapy was required in 11 CTA and 3 CA patients, while testosterone therapy in 2 AA and 4 CTA patients. Substitution therapy with L-thyroxine was used in 30 acromegaly patients: 2 in the AA group, 20 in the CTA group, 8 in the CA group, and 10 controls. In the acromegaly group, 23 patients were supplemented with vitamin D and 8 with calcium. In the CG, 3 of the patients received vitamin D, and 2 received calcium supplementation. Three patients with acromegaly received anti-resorptive therapy bisphosphonate). All participants signed a written informed consent. The local Bioethics Committee approved the protocol of the present study. The research was conducted in accordance with the Declaration of Helsinki.

We measured weight (kg) and height (m) to calculate body mass index (BMI) of the patients. Fasting venous blood samples were obtained from all participants. The GH and IGF-1 concentrations were measured using chemiluminescence immunoassay (Immulite 2000, Siemens Healthcare Diagnostics, USA). IGF-1 reference ranges were sex- and age-matched. The SCL concentration was measured using sandwich ELISA (Biomedica Medizinprodukte GmbH, Austria). The lower limit of quantification (LLOQ) was <7.5 pmol/l. The lower limit of detection (LOD) was 3.2 pmol/l. Intra-assay precision was ≤ 7%, inter-assay precision was ≤ 10%. The OPG concentration was measured using sandwich ELISA (Biomedica Medizinprodukte GmbH, Austria). The lower limit of quantification (LLOQ) was <0.08 pmol/l. The lower limit of detection (LOD) was 0.07 pmol/l. Intra-assay precision was ≤ 4%, inter-assay precision was ≤ 3%. The RANK-L concentration was measured using sandwich ELISA (Biomedica Medizinprodukte GmbH, Austria). The lower limit of quantification (LLOQ) was 0.008 pmol/l. The lower limit of detection (LOD) was 0.01 pmol/l. Intra-assay precision was ≤ 3%, inter-assay precision was ≤ 5%. Serum samples were stored at -70°C.

The BMD of the lumbar spine (LS; L1–L4) and femoral neck (FN) was measured using the dual-energy X-ray absorptiometry (DXA) with Hologic – Discovery QDR Series densitometer. The results were expressed as BMD (g/cm2), T-score, and Z-score.

Statistical analysis was performed using R for Windows software (version 3.5). Variables were presented as mean, standard deviation (SD), median, and interquartile ranges (IQR). The Shapiro–Wilk’s test, histograms, and Q-Q plots were used to determine the normality of the data distribution. Mann-Whitney U test (first classification) or Kruskal-Wallis test (second and third classification) were applied to compare quantitative variables between studied groups. Frequency distributions were analyzed by Fisher’s exact test. Correlations were calculated using Spearman’s rank correlation test. P-values less than 0.05 were considered statistically significant.

## 3 Results

The general characteristics of the acromegaly patients and the control group are shown in **Table 1**. We found no statistically significant differences in age, sex, weight, height, and BMI among studied groups, regardless of applied classification (p>0.05).

**Table 1:** General characteristics of the acromegaly and control groups.

		AA	CTA	CA	CG	AA+CTA+CA	CTA+CA
**Age (years)**	mean	60.11	61.80	57.08	56.67	60.01	60.00
	SD	10.98	11.07	11.23	10.90	11.16	11.28
	median	63.00	63.00	59.00	59.00	62.50	62.00
	IQR	51.00; 69.00	55.00; 69.50	46.25; 65.00	49.00; 64.75	52.75; 69.00	53.00; 68.50
**Height (m)**	mean	173.44	167.38	166.79	168.54	167.94	167.16
	SD	13.56	9.47	6.90	8.55	9.41	8.53
	median	170.00	175.00	165.50	174.75	166.00	166.00
	IQR	164.0; 176.00	160.0; 175.00	162.0; 170.25	167.5; 174.75	160.00; 175.00	160.00; 174.50
**Body mass (kg)**	mean	92.22	81.41	81.65	78.90	82.84	81.50
	SD	13.60	16.19	17.86	15.23	16.65	16.70
	median	95.00	84.00	74.50	78.00	82.50	78.00
	IQR	84.00; 104.00	8.00; 95.50	70.00; 92.50	67.00; 87.25	70.00; 97.25	68.50; 95.50
**BMI (kg/m^2^)**	mean	30.83	29.12	28.70	27.63	29.20	28.96
	SD	4.90	4,53	6,30	4.08	5.19	5.23
	median	27.90	29.00	27,89	27.41	28.15	28.20
	IQR	27.12; 35.20	26.17; 32.50	24.95; 31.05	24.84; 29.40	25.58; 32.51	25.26; 31.81
IGF-1 (ng/ml)^*^	mean	317.00	134.91	147.46	112.44	161.85	139.69
	SD	144.52	37.96	54.92	28.38	87.30	45.17
	median	307.00	125.00	140.00	112.50	140.00	133.00
	IQR	218.00; 412.00	102.50; 159.00	111.75; 170.00	93.45; 131.75	106.00; 186.25	102.50; 163.00
GH (nadir) (ng/ml)^**^	mean	3.35	1.39	1.93	0.55	1.81	1.59
	SD	2.58	1.72	2.57	0.69	2.21	2.08
	median	2.59	1.02	0.74	0.28	1.09	0.88
	IQR	1.73; 4.47	0.52; 1.60	0.52; 1.60	0.11; 0.83	0.46; 2.25	0.44; 1.72

BMI, body mass index; SD, standard deviation; IQR, interquartile ranges; AA, active acromegaly; CTA, controlled acromegaly; CA, cured acromegaly; CG, control group; ^*^AA vs CG (p<0.001), AA vs CTA (p<0.001), AA vs CA (p=0.004), CTA vs CG (p=0.024), CA vs CG (p=0.009), AA+CTA+CA vs CG (p<0.001), AA vs CTA+CA (p<0.001), CTA+CA vs CG (p<0.001); ^**^AA vs CG (p<0.001), AA vs CTA (p<0.025), AA vs CA (p=0.017), CTA vs CG (p<0.001), CA vs CG (p=0.002), AA+CTA+CA vs CG (p<0.001), AA vs CTA+CA (p<0.001), CTA+CA vs CG (p<0.001).

Significant differences in IGF-1 concentrations were revealed between the following groups: AA vs CG (p<0.001), AA vs CTA (p<0.001), AA vs CA (p=0.004), CTA vs CG (p=0.024), CA vs CG (p=0.009). Furthermore, significantly higher IGF-1 concentrations were shown in patients with acromegaly in comparison with CG for both classification I (AA+CTA+CA, CG; p<0.001) and classification II (AA, CTA+CA, CG; p<0.001) (**Table 1**).

Significant differences in GH concentrations were shown between the following groups: AA vs CG (p<0.001), AA vs CTA (p<0.025), AA vs CA (p=0.017), CTA vs CG (p<0.001), CA vs CG (p=0.002). GH concentrations were significantly higher in groups of patients with acromegaly than in CG for both classification I (AA+CTA+CA, CG; p<0.001) and classification II (AA, CTA+CA, CG; p<0.001) (**Table 1**).

**Figure 1:**
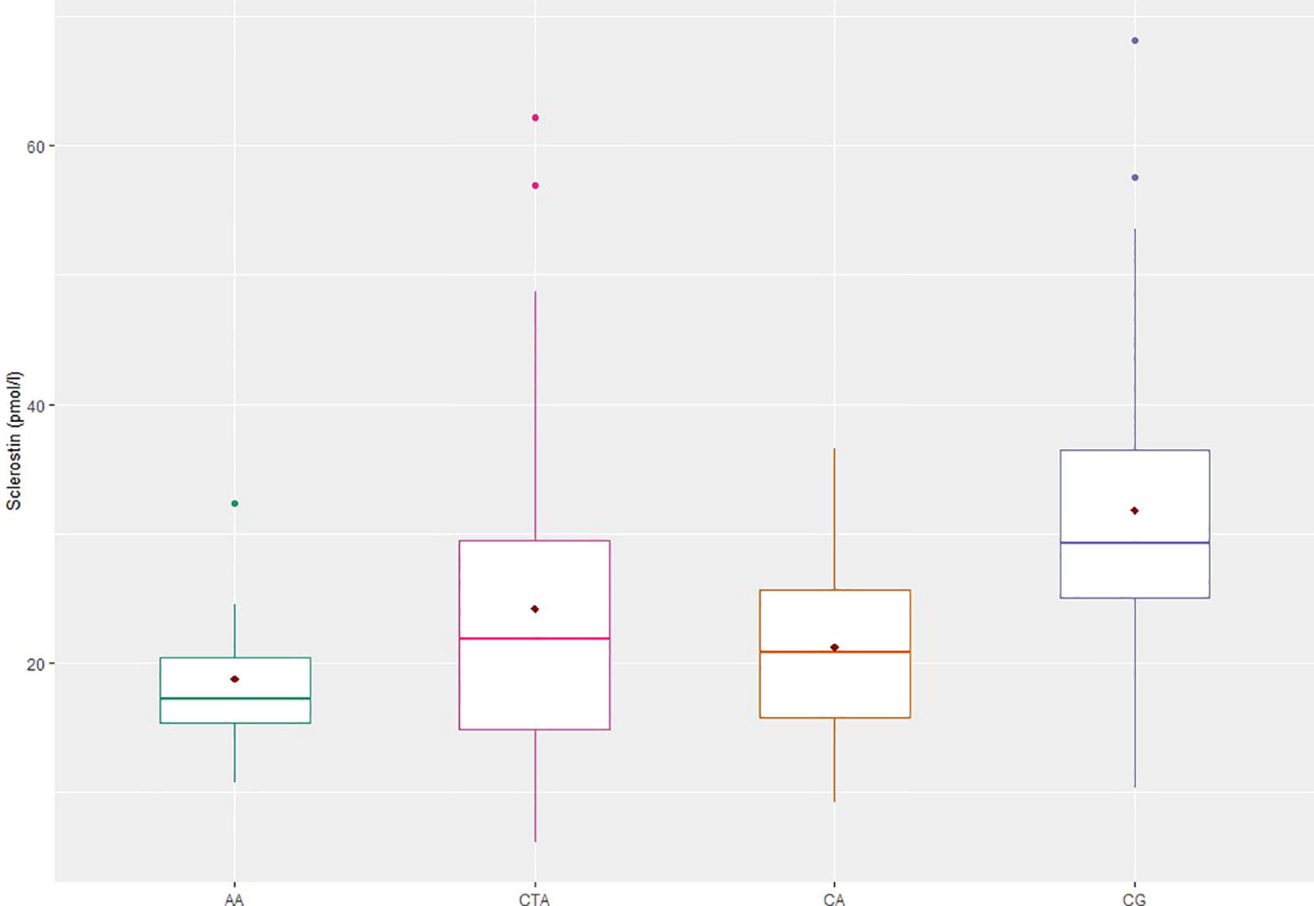
Sclerostin concentrations in patients with acromegaly and controls. AA, active acromegaly; CTA, controlled acromegaly; CA, cured acromegaly; CG, control group; AA vs CG, CTA vs CG, CA vs CG; p < 0.001.

The highest SCL concentration was observed in the CG and the lowest in the AA group (**Figure 1**). Significantly lower sclerostin concentrations were found in patients with acromegaly in comparison with CG, regardless of used classification (AA, CTA, CA, CTA+CA, AA+CTA+CA vs CG: p < 0.001, respectively) (**Table 2**).

**Table 2:** Bone metabolism parameters and BMD in patients with acromegaly and controls.

		AA	CTA	CA	CG	AA+CTA+CA	CTA+CA
**SCL (pmol/l)^#^**	mean	18.75	24.18	21.19	31.81	22.56	23.04
	SD	6.89	12.66	7.19	11.22	10.61	10.93
	median	17.19	21.80	20.78	29.22	21.54	21.78
	IQR	15.41; 20.37	14.86; 29.50	15.78; 25.70	25.00; 36.46	14.98; 27.85	14.98; 28.17
**OPG (pmol/l)^^^**	mean	4.42	5.57	4.08	3.73	4.94	5.00
	SD	1.04	1.90	1.35	1.12	1.78	1.85
	median	4.67	5.18	4.00	3.59	4.89	4.93
	IQR	3.75; 5.20	4.32; 6.38	3.01; 4.91	2.94; 4.67	3.82; 5.61	3.82; 5.61
**RANK-L (pmol/l)**	mean	0.17	0.15	0.16	0.14	0.16	0.15
	SD	0.10	0.12	0.0	0.10	0.10	0.11
	median	0.14	0.13	0.16	0.11	0.14	0.14
	IQR	0.09; 0.24	0.07; 0.19	0.09; 0.20	0.09; 0.19	0.09; 0.20	0.08; 0.20
**LS BMD (g/cm^2^)**	mean	1.05	0.99	1.01	0.98	1.00	1.00
	SD	0.16	0.16	0.19	0.14	0.17	0.17
	median	1.04	0.98	0.96	0.99	0.98	0.98
	IQR	0.91; 1.21	0.86; 1.12	0.93; 1.12	0.89; 1.07	0.89; 1.13	0.88; 1.12
**FN BMD (g/cm^2^)**	mean	0.87	0.80	0.86	0.82	0.83	0.82
	SD	0.22	0.12	0.15	0.14	0.15	0.13
	median	0.83	0.81	0.84	0.81	0.82	0.82
	IQR	0.69; 1.01	0.71; 0.88	0.75; 0.94	0.72; 0.92	0.72; 0.92	0.73; 0.92

SCL, sclerostin; OPG, osteoprotegerin; RANK-L, receptor activator for nuclear factor κ B ligand; BMD, bone mineral density; LS, lumbar spine; FN, femoral neck; SD, standard deviation; IQR, interquartile ranges; AA, active acromegaly; CTA, controlled acromegaly; CA, cured acromegaly; CG, control group; ^#^AA, CTA, CA, CTA+CA, AA+CTA+CA vs CG: p < 0.001, respectively; ^^^CTA vs CA (p=0.002), CTA vs CG (p<0.001), CTA+CA vs. CG (p<0.001), AA+CTA+CA vs. CG (p<0.001).

Significant differences in OPG concentrations were revealed between the following groups: CTA vs CA (p=0.002) and CTA vs CG (p<0.001). Focusing on the first and second classifications, statistically significant differences in OPG concentrations were also found in the following groups: CTA+CA vs. CG (p<0.001) and AA+CTA+CA vs. CG (p<0.001) (**Table 2**).

Similarly, no significant differences in RANK-L concentrations were found between the groups, regardless of the adopted classification (p>0.05) (**Table 2**).

There were no significant differences in LS and FN BMD between studied groups for all applied classifications (p>0.05) (**Table 2**). Differences in LS Z-score values were statistically significant only between the AA+CTA+CA group and the CG (p=0.046). We found no significant differences in FN T-score, FN Z-score, and LS T-score (p>0.05) (data not shown).

We did not find statistically significant correlations between SCL and GH or IGF-1 in any of the analyzed groups (p>0.05).

**Figure 2:**
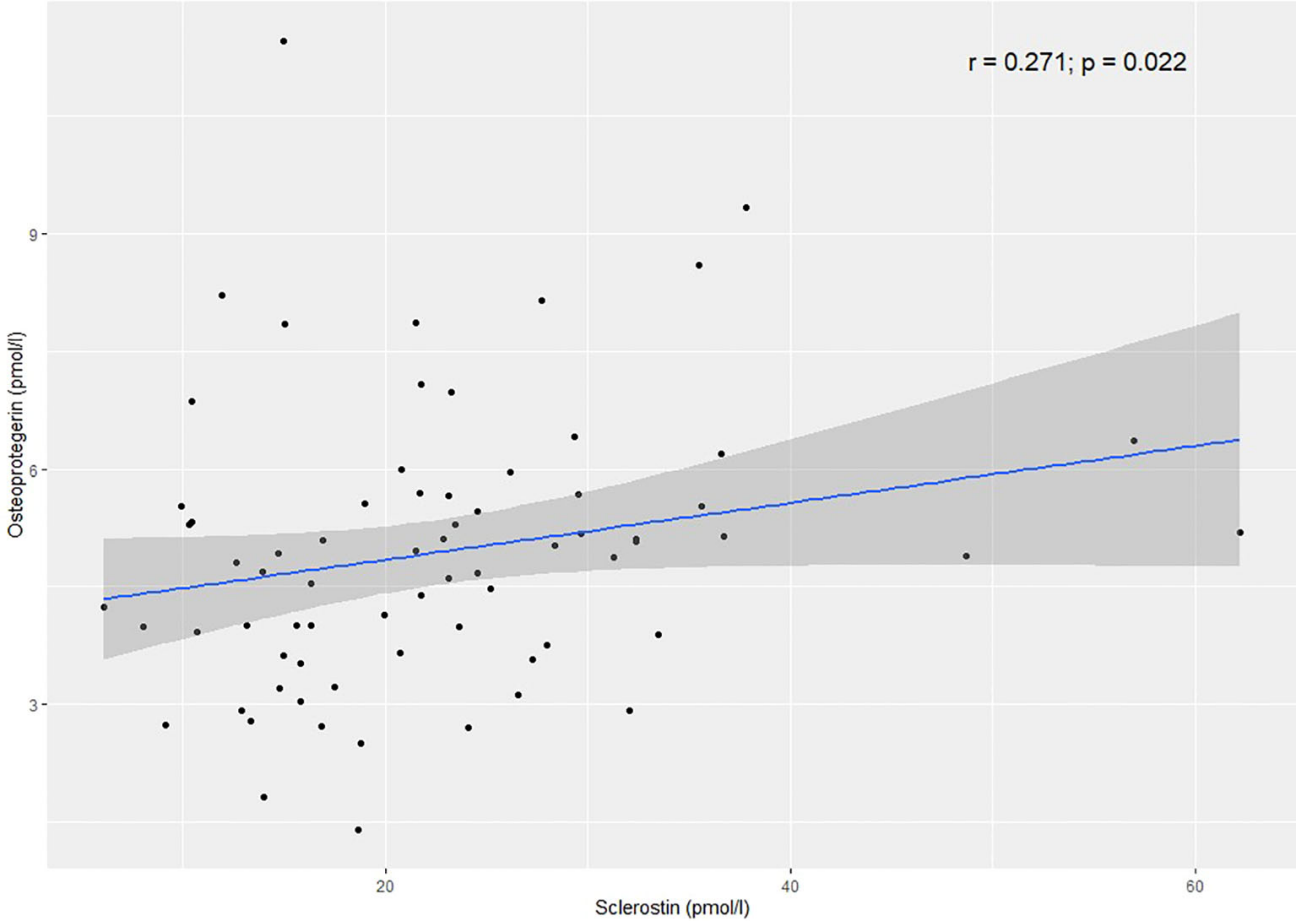
Correlation between sclerostin and osteoprotegerin in the AA+CTA+CA group.

**Figure 3:**
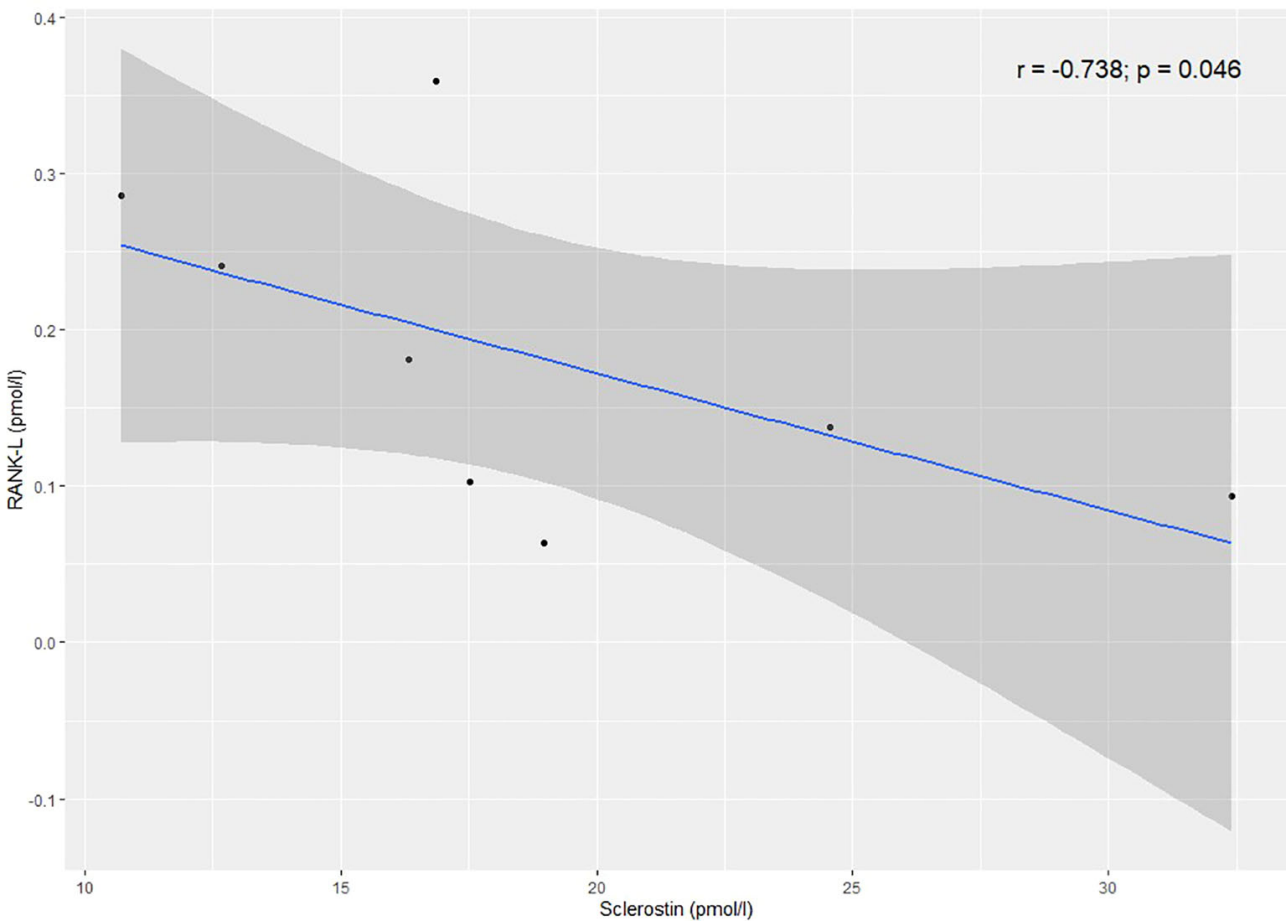
Correlation between sclerostin and RANK-L in the AA group.

In the AA+CTA+CA group, there was a statistically significant positive correlation between SCL and OPG (r=0.271; p=0.022) (**Figure 2**). A significant negative correlation was found between SCL and RANK-L in the AA group (r=-0.738; p=0.046) (**Figure 3**).

There were no correlations between SCL and BMD, T-score, Z-score at the lumbar spine and femoral neck (p>0.05).

SCL concentration correlated positively with age in the following groups: CA (r=0.448, p=0.028), CTA (r=0.466 p=0.003), CTA+CA (r=0.446, p<0.001), and AA+CTA+CA (r=0.425, p<0.001).

We found a significant negative correlation between OPG and GH in the CA group (r=-0.431, p=0.037). In the AA+CTA+CA and CTA+CA groups, there were significant negative correlations between OPG and IGF-1 (r=-0.283, p=0.017; r=-0.289, p=0.022, respectively).

A statistically significant negative correlation between RANK-L and GH was observed in the CTA group (r=-0.328, p=0.041). In the CA, CTA+CA, AA+CTA+CA groups, we found significant positive correlations between RANK-L and IGF-1 (r=0.543, p=0.006; r=0.367, p=0.003, r=0.371, p=0.001, respectively). A correlation between RANK-L and IGF-1 in the AA group was close to the threshold of statistical significance (r=0.683, p=0.05).

Significant negative correlations between OPG and RANK-L were present in the following groups: CTA (r=-0.456, p=0.004), CA (r=-0.597, p=0.002), CTA+CA (r=-0.494, p<0.001), AA+CTA+CA (r=-0.506, p<0.001), and CG (r=-0.372, p=0.006).

Statistically significant correlations between OPG/RANK-L concentrations and BMD parameters in patients with acromegaly and controls are presented in **Table 3**.

**Table 3:** Statistically significant correlations between OPG/RANK-L concentrations and BMD parameters in patients with acromegaly and controls.

	Groups	R	p
**OPG (pmol/l) and LS BMD (g/cm^2^)**	**CTA**	-0.325	**0.046**
**OPG (pmol/l) and FN BMD (g/cm^2^)**	**CTA**	-0.491	**0.002**
	**CTA+CA**	-0.399	**0.001**
	**AA+CTA+CA**	-0.354	**0.003**
**OPG (pmol/l) and FN T-score**	**CTA**	-0.460	**0.004**
	**CTA+CA**	-0.435	**<0.001**
	**AA+CTA+CA**	-0.395	**<0.001**
**OPG (pmol/l) and FN Z-score**	**CG**	0.433	**0.001**
**RANK-L (pmol/l) and LS BMD (g/cm^2^)**	**CTA+CA**	0.258	**0.045**
	**AA+CTA+CA**	0.253	**0.035**
**RANK-L (pmol/l) and FN BMD (g/cm^2^)**	**CTA**	0.526	**0.001**
	**CA**	0.444	**0.033**
	**CTA+CA**	0.513	**<0.001**
	**AA+CTA+CA**	0.450	**<0.001**
**RANK-L (pmol/l) and FN T-score**	**CTA**	0.498	**0.001**
	**CA**	0.419	**0.047**
	**CTA+CA**	0.499	**<0.001**
	**AA+CTA+CA**	0.437	**0.001**
**RANK-L (pmol/l) and FN Z-score**	**CTA**	0.398	**0.013**
	**CTA+CA**	0.388	**0.002**
	**AA+CTA+CA**	0.308	**0.010**

OPG, osteoprotegerin; RANK-L, receptor activator for nuclear factor κ B ligand; BMD, bone mineral density; LS, lumbar spine; FN, femoral neck; AA, active acromegaly; CTA, controlled acromegaly; CA, cured acromegaly; CG, control group.

## 4 Discussion

In the present study, SCL concentration was lower in patients with acromegaly compared to the controls. In our opinion, consistent with the previously described theory, a reduction in SCL concentration could be a compensatory mechanism to counteract enhanced bone fragility in acromegaly and a consequent increase in vertebral fracture risk (16). The lack of differences in SCL concentration between groups of patients with acromegaly may indicate a persistent disturbance of SCL metabolism despite proper disease control or successful treatment of patients. Findings from previous studies showed that increased vertebral fracture risk is also observed in patients with biochemically controlled acromegaly (4, 30). A different reason for lower SCL in acromegaly may be osteocyte dysfunction, which results in reduced synthesis of sclerostin. The literature describes a positive correlation of SCL concentration with the density, number, and thickness of bone trabeculae, which suggests that increased trabecular bone resorption in osteoporotic bone is associated with a lower number of mature osteocytes secreting SCL (31). Some authors suggest that low SCL levels might be associated with low BMD, and thus increased mechanical load on the bones (32). However, the present study did not show significant correlations between SCL and BMD. Since the body composition changes in acromegaly with an increase in lean mass and a decrease in fat mass, Chen et al. hypothesize that an increase in lean mass results in mechanical loading and subsequently, a decline in SCL concentration in these patients (16). Silva et al., in a study on premenopausal women with acromegaly without any pituitary deficiency, also demonstrated that SCL levels were lower in the active acromegaly group compared with the healthy control group (18). However, some research groups have observed no significant difference between acromegaly patients and controls, and even one study showed increased sclerostin levels in active acromegaly (17, 20, 21). The reasons for the discrepancies between the studies may be explained by differences in assays and characteristics of the study populations in terms of age, sex, body composition, physical activity, and the duration of biochemical remission and active disease (16, 19).

In our study, we found no direct interaction between the GH/IGF-1 axis and SCL. Most studies conducted on groups of patients with acromegaly have not confirmed a direct influence of the GH/IGF-1 axis on sclerostin concentration, however, both positive and negative relationships have been observed (16–20). Ardawi et al. suggested that in premenopausal women, the anabolic effect of physical activity on bone may be achieved by reducing the concentration of SCL and increasing the concentration of IGF-1 (33). A negative correlation was also found in postmenopausal women with type 2 diabetes (34). However, similar to the results obtained in the present research, in a study involving men over 65 years of age, no relationship was shown between SCL concentration and IGF-1 concentration, using three different analytical kits to determine SCL concentration (35).

The current literature lacks data related to the association of SCL with the OPG/RANK-L system in acromegaly. Available studies show that SCL, besides its influence on the bone formation process, regulates bone resorption (9). The activation of the Wnt/β-catenin pathway in osteoblasts results in increased OPG expression and reduction of bone resorption. SCL, as an inhibitor of this pathway, contributes to the decrease of the OPG/RANK-L ratio, thus activating osteoclastogenesis (36). An *in-vitro* study showed that SCL, in a dose-dependent matter, increases the expression of RANK-L mRNA and decreases the expression of OPG mRNA (14). Consistent results were presented by Tu et al. in a study on mice (37). However, in a different study using a mouse model, the researchers found no influence of anti-SCL antibodies on OPG and RANK-L levels (13). The current study revealed a significant negative correlation between SCL and RANK-L in the AA group and a significant positive correlation between SCL and OPG in the AA+CTA+CA group. We found no significant correlations in other groups. Similarly, in one study, there was a positive correlation between SCL and OPG in both controls and subjects with fractures (38). To further understand associations between SCL and OPG/RANK-L system, more studies, especially *in vivo*, are needed. It would be an appealing research direction in patients with acromegaly, but also patients with other skeletal disorders and healthy individuals.

In the present study, no statistically significant correlation was found between SCL concentration and BMD. The obtained results are consistent with two previous studies on patients with acromegaly (16, 19). In a population study involving over 1,800 pre- and postmenopausal women, a negative correlation was observed between SCL concentration and BMD at the femoral neck and lumbar spine in both age groups, but after adjusting for age and BMI, this association was not significant (39). In contrast, Garnero et al. showed a weak, positive correlation between SCL concentration and BMD in the spine and total hip in postmenopausal women (40). A similar correlation was described in other studies of both women and men (32, 38, 41, 42). The researchers suggest that higher SCL concentration may result from higher bone mass and the number of osteocytes or may be related to mechanical stress on the bones, which, as studies show, affects SCL synthesis (9, 32, 43). Inconsistent findings may be due to different study populations and the influence of many factors regulating SCL concentration and affecting BMD, such as age, BMI, hormones, and other molecules’ concentrations (33, 44).

The present study showed a positive association between SCL concentration and age in the CTA, CA, AA+CTA+CA, and CTA+CA groups. These results are consistent with data from previous studies (33, 42–44). Researchers postulate that the decline in estrogen levels with age in women, but also in men, may be responsible for the increase in SCL concentration with age (44, 45). Other studies have shown that, regardless of age and gender, SCL levels correlate inversely with the glomerular filtration rate (GFR), therefore, progressive renal dysfunction over time may partially explain the increase in SCL levels (45). Furthermore, higher SCL levels in the elderly, compared to the younger individuals, may result from a sedentary lifestyle and lower physical activity, which is associated with a lower mechanical load on the bones and thus is responsible for the stimulation of SCL synthesis (33, 43).

It is well known that the OPG/RANK-L system plays a crucial role in the regulation of osteoclastogenesis and, thus, in the remodeling of bone tissue. RANK-L stimulates bone resorption, whereas OPG, a soluble decoy receptor of RANK-L, prevents bone resorption (46). Moreover, studies in mice suggest that OPG also inhibits the release of RANK-L from osteoblasts (23, 47). Additionally, OPG affects the half-life of the RANK-L, and RANK-L controls the internalization and biodegradation of OPG (48). It is worth mentioning that during the differentiation and maturation of osteoblasts, there is an increase in OPG mRNA and a decrease in RANK-L mRNA. Gori et al. suggest that changes in the RANK-L/OPG ratio during osteoblastogenesis contribute to the coordination of subsequent cycles of bone formation and bone resorption in the bone remodeling process (49). We found an inverse association between OPG and RANK-L levels in most of the studied groups of patients with acromegaly (CTA, CA, CTA+CA, AA+CTA+CA) and CG. The obtained results are consistent with the mechanisms of action of both molecules. It should be noted that we used assays for the soluble form of the RANK-L, but OPG also affects the availability of membrane-bound RANK-L (23).

The present study showed an increased OPG level in patients with acromegaly, particularly in the CTA group, which might be a response to a persistent bone resorption process. The confirmation of this hypothesis is the fact that patients with well-controlled activity of disease still have increased fracture risk (30). Valassi et al. also demonstrated that osteoprotegerin levels were significantly higher in patients with controlled acromegaly as compared with controls (21). No significant differences in OPG levels between the CA and CG groups, and significantly higher OPG in the CTA group compared with the CA group might indicate the restoration of bone remodeling balance in successfully treated patients at our Clinic. It is worth noting that IGF-1 and GH affect OPG synthesis differently. According to prior studies, IGF-1 decreases, and GH increases OPG levels (27, 28). Depending on the severity of the GH/IGF-1 system imbalance, each of the mechanisms might play a more important role. Apparently, OPG blood levels increase when the disease is well-controlled. Ueland et al. conducted a study on several groups of patients, including 24 patients with active acromegaly without previous treatment and 16 patients with GH deficiency. OPG levels in both groups did not differ compared to healthy controls (50). Constantin et al. did not observe differences in OPG and RANK-L levels between patients with active acromegaly and patients with nonfunctioning pituitary adenomas. Moreover, the RANK-L/OPG ratio did not change after pharmacological or surgical treatment of acromegaly. The authors suggest that the lack of changes in OPG and RANK-L concentrations following treatment might be caused by the fact that both OPG and RANK-L are produced not only in bones, but also in other tissues, and their blood levels might not reflect their synthesis in bone (51). The short observation period in the study (3-6 months) might also influence the results. In other study on 31 patients with acromegaly, there were no differences with regard to OPG, RANK-L, and RANK-L/OPG ratio compared with the control group (52). Our results, consistent with the studies discussed above, also did not show significant differences in RANK-L levels between the study groups. Only one recently published study demonstrated that RANK-L levels were significantly higher in the acromegaly group compared to the healthy controls (53).

It is known that active acromegaly is characterized by increased bone turnover, but the mechanism responsible for the activation of osteoclastogenesis has not been fully understood. Constantin et al. did not observe an association between IGF-1 levels and RANK-L/OPG ratio in patients with acromegaly (51). In our study, however, we found significant negative correlations between IGF-1 and OPG in the AA+CTA+CA and CTA+CA groups. IGF-1 and RANK-L correlated positively in the AA+CTA+CA, CTA+CA, AA, and CA groups. The results are similar to the ones presented in a population study on 500 healthy Chinese women (54). These results suggest that the influence of IGF-1 on bone resorption might be exerted through the regulation of the OPG/RANK-L system (54). On the other hand, in a study on 80 Korean men aged 42-70 years, no correlations between IGF-1 and OPG/RANK-L were found, which can be explained by the influence of sex hormones on the OPG/RANK-L system (55).

The literature offers scarce published data on GH’s influence on OPG levels. An *in-vitro* study showed that GH induces the synthesis of OPG (28). This is in keeping with iv vivo studies on patients with GH deficiency (56, 57). To the best of our knowledge, this is the first study that investigates the association between GH and OPG/RANK-L system in patients at different stages of acromegaly activity. In the present study, we found a negative correlation between GH levels and OPG levels in the CA group. GH levels and RANK-L levels correlated negatively in the CTA group. Regarding the anabolic influence of GH on bone, we might consider whether GH inhibits osteoclastogenesis by decreasing RANK-L availability. Interactions between GH/IGF-1 and OPG/RANK-L system are complex and probably change in time, depending on how effectively the acromegaly is controlled and on other regulating mechanisms.

Many authors have analyzed the impact of the components of the OPG/RANK-L system on BMD, but their results are inconsistent. The discrepancies may be caused by various factors, including the use of different tests to determine OPG concentration and polymorphism of the gene encoding OPG (58, 59). Ozer et al. demonstrated that neither the RANK-L nor RANK-L/OPG ratio were correlated with FN BMD or LS BMD. Furthermore, they presented an inverse correlation between OPG and FN BMD in patients with acromegaly (52). Similarly, we observed a negative correlation between OPG concentration and LS BMD in the CTA group and between OPG and FN BMD in the AA+CTA+CA, CTA+CA, and CTA groups. Moreover, there was a significant positive correlation between the RANK-L and LS BMD in the AA+CTA+CA and CTA+CA groups and between the RANK-L and FN BMD in the AA+CTA+CA, CTA+CA, CTA, and CA groups. The presented associations between OPG/RANK-L system and BMD seem understandable, considering the mechanism of action of these proteins in bone tissue. When in a state of reduced BMD associated with increased bone turnover, OPG concentration increases, constituting a protective mechanism against further bone loss. In turn, with increased BMD, the OPG concentration decreases, which improves the availability of RANK-L and leads to the activation of osteoclastogenesis. Consistent with this hypothesis, a study involving women with osteoporosis and accelerated bone turnover showed higher OPG concentrations in osteoporotic women than in women of a similar age without osteoporosis (59). The results presented in this study suggest that the OPG/RANK-L system takes part in bone metabolism regulation and affects BMD in patients with acromegaly, especially at the femoral neck.

There were some limitations to our study. First, since acromegaly is a rare disease, the study sample of patients with active acromegaly was inevitably small. Most of the included participants are under constant supervision, which contributes to the efficient monitoring and treatment of these patients. Second, the duration and complications of the disease, comorbidities, and applied treatment, physical activity may influence the results. In the current study, to assess the influence of the disease activity on the analyzed parameters, we used additional classifications of the participants. Another strength of the study is the creation of the database by one researcher.

In conclusion, SCL concentration was lower in patients with acromegaly compared to the controls, regardless of the acromegaly activity. There may be several reasons for the reduction in SCL levels. However, we postulate that the most likely cause is negative feedback to accelerated bone loss, thus enhanced bone fragility and increased risk of vertebral bone fractures. The assessment of interactions between SCL and OPG/RANK-L system in acromegaly continues to be challenging due to many factors influencing levels of SCL, OPG, and RANK-L, however, it is worth noting that a more detailed understanding of the pathomechanism of bone complications in acromegaly may contribute to better diagnostic and therapeutic management and improvement of the quality of life in acromegaly patients. OPG/RANK-L system also plays a role in bone turnover in acromegaly. OPG levels are increased in acromegaly, which may reflect a compensatory mechanism to increased bone resorption. Higher OPG levels in the CTA group than CG may suggest, that an imbalance between bone resorption and bone formation exists even in well-controlled acromegaly patients. There is an association between the OPG/RANK-L system and BMD, mainly FN BMD, which in the future may allow the usage of OPG as a marker of bone turnover in acromegaly. However, there is a need for further studies to confirm these results, especially in active acromegaly.

## Data Availability

The raw data supporting the conclusions of this article will be made available by the authors, without undue reservation.
